# Polymer Blends for Improved CO_2_ Capture Membranes

**DOI:** 10.3390/polym11101662

**Published:** 2019-10-12

**Authors:** Alireza Zare, Lorenza Perna, Adrianna Nogalska, Veronica Ambrogi, Pierfrancesco Cerruti, Bartosz Tylkowski, Ricard García-Valls, Marta Giamberini

**Affiliations:** 1 Department of Chemical Engineering, Universitat Rovira I Virgili, Av. Països Catalans, 26, 43007 Tarragona, Spain; alireza.zare@urv.cat (A.Z.); lorenza.perna@hotmail.com (L.P.); ricard.garcia@urv.cat (R.G.-V.); 2 Department of Chemicals, Materials and Production Engineering, University of Naples Federico II, Piazzale Tecchio 80, 80125 Naples, Italy; ambrogi@unina.it; 3 Chemistry Technology Centre of Catalonia (CTQC), C/Marcel·lí Domingo, 43007 Tarragona, Spain; adrianna.nogalska@ctqc.org (A.N.); bartosz.tylkowski@ctqc.org (B.T.); 4 Eurecat, Centre Tecnològic de Catalunya, C/Marcel·lí Domingo, 43007 Tarragona, Spain; 5 Institute of Polymers, Composites and Biomaterials, National Research Council, Via Campi Flegrei 34, 80078 Pozzuoli, Italy; cerruti@ipcb.cnr.it

**Keywords:** carbon dioxide capture, polyethylene imine, polysulfone, artificial stomata

## Abstract

We investigated the possibility of improving the performance of polysulfone (PSf) membranes to be used in carbon dioxide capture devices by blending PSf with a commercial polyethylene imine, Lupasol G20, previously modified with benzoyl chloride (mG20). Additive amount ranged between 2 and 20 wt %. Membranes based on these blends were prepared by phase inversion precipitation and exhibited different morphologies with respect to neat PSf. Surface roughness, water contact angles, and water uptake increased with mG20 content. Mass transfer coefficient was also increased for both N_2_ and CO_2_; however, this effect was more evident for carbon dioxide. Carbon dioxide absorption performance of composite membranes was evaluated for potassium hydroxide solution in a flat sheet membrane contactor (FSMC) in cross flow module at different liquid flow rates. We found that, at the lowest flow rate, membranes exhibit a very similar behaviour to neat PSf; nevertheless, significant differences can be found at higher flow rates. In particular, the membranes with 2 and 5 wt % additive behave more efficiently than neat PSf. In contrast, 10 and 20 wt % additive content has an adverse effect on CO_2_ capture when compared with neat PSf. In the former case, a combination of additive chemical affinity to CO_2_ and membrane porosity can be claimed; in the latter case, the remarkably higher wettability and water uptake could determine membrane clogging and consequent loss of efficiency in the capture device.

## 1. Introduction

Since the late nineteenth century, rapid economic growth has provoked a huge increase in energy production and consumption. Given that energy has largely depended on the use of fossil fuels, their abundant use has turned into a big concern due their adverse effects on environment, in particular, carbon dioxide emission. CO_2_ emissions contribute to 60% of global warming as well as the greenhouse (GHG) effect [1]. According to the Emission Database for Global Atmospheric Research, global emissions from carbon dioxide have been increasing, rising by approximately 50% in only two decades. In 2016, carbon dioxide concentration reached the record value of 402 ppm [2], which has led to an increase of global surface temperature of about 0.8 °C. The intergovernmental Panel on Climate Change (IPCC) Fifth Assessment Report, issued in 2013, indicated that the temperature rise should be kept below 2 °C relative to pre-industrial levels to prevent the worst effects of climate change; this has inevitably encouraged a strict control of carbon dioxide emissions. To reduce CO_2_ concentration in the environment, several approaches are possible: promoting energy conservation, increasing the use of low carbon fuels (hydrogen, natural gas, or nuclear power), deploying renewable energy (solar, wind, hydropower, and bio-energy), and eventually capturing and storing carbon dioxide itself. The last approach could allow the subsequent use of the captured gas as a carbon source; nevertheless, this implies that the carbon dioxide must first be captured at atmospheric pressure and stored efficiently. Current processes are based on physical or chemical solvents as well as membrane technology; recently, new hybrid processes have been developed, which combine advanced membrane techniques with an effective CO_2_ absorption process in one device, called a contactor [3,4,5]. In these compact systems, an advanced gas membrane, which helps the mass exchange process by increasing the surface area for phase contact, is present between the gas and an absorbing aqueous solution [3,6]. This approach is inspired by natural photosynthesis, in which leaves, by means of stomata, are able to efficiently capture carbon dioxide. In the case of membrane contactors, pores act as stomata. This system offers additional advantages: it is not necessary to disperse one phase into another due to the large contact surface between the phases. It is also a small modular system, which can be easily assembled and integrated in other devices. The system is not sensitive to flooding, channeling, or back-mixing; it can also be operated in a wide range of flow rates.

Recently, we reported a biomimetic contactor designed as artificial stomata [7]. This device takes advantage of a polysulfone membrane in combination with a potassium hydroxide solution. Different membrane contactors, with morphologies ranging from spongy-like to open microvoids, were prepared by phase inversion precipitation and different membrane preparation parameters. The highest CO_2_ absorption flux (i.e., 67.5 mmol/m^2^ s) was found in the case of a fingerlike macrovoids membrane, in which CO_2_ absorption was even higher than in natural stomata (40 µmol/m^2^ s). This is also the highest value reported for an artificial system.

Amines and aminic compounds have found wide applications as CO_2_ removing agents due to the reversible reaction between aminic groups (basic) and CO_2_ (acidic). Amine carriers can be divided into unhindered and hindered. In the former case, CO_2_ transport occurs via a zwitterion formation, which is subsequently quickly deprotonated by another amine group, producing a carbamate ion and a protonated amine. Under this mechanism, 0.5 mol carbon dioxide can be loaded per amine group [7,8]. On the other hand, when hindered amines are considered, the presence of a bulky group directly linked to the amine determines carbamate instability. Therefore, carbon dioxide is carried as a carbonate group in the presence of water and the maximum loading in this case is one CO_2_ molecule per amine group. Moreover, in the case of hindered amines, the overall reaction rate is higher [9]. For this reason, hindered amines are considered good carriers in carbon dioxide-facilitated transport membranes. Amine carriers have been reported both as mobile and fixed-site: the second option could be preferable since mobile carriers are not covalently linked to the polymeric membrane and can produce a leakage phenomenon in a relatively easy way [10]. Recently, nanocomposite membranes based on polyvinylamine (PVAm)/piperazine glycinate (PG) for CO_2_ removal were reported [11], which showed high CO_2_/N_2_ selectivity and good CO_2_ permeance. Zi Tong et al. [10] investigated several sterically hindered polyvinylamines with different degrees of steric hindrance and found significant improvement in CO_2_ permeance and CO_2_/N_2_ selectivity in comparison with unhindered polyvinylamine.

In this paper, our aim is to increase the performance of the polysulfone membrane contactor by blending commercial PSf with a commercial, hyperbranched polyethylene imine, specifically, Lupasol G20. The presence of the basic nitrogen atoms should increase the membrane affinity for carbon dioxide, thus improving its capture and permeability with respect to PSf. On the other hand, Lupasol G20 is a water-soluble polymer; this could prevent membrane preparation from a water coagulation bath as previously described for neat PSf as well as membrane stability in contact with the storing aqueous solution. For this reason, before blending with PSf, Lupasol G20 was partially chemically modified by means of benzoyl chloride. Additionally, chemical modification of Lupasol G20 is expected to preferably occur on the unhindered amines, converting them to hindered ones. The loading capability of the modified Lupasol is expected to improve as a consequence of this reaction. Subsequent blending of the modified Lupasol with PSf was performed in small amount (i.e., between 2 and 20 wt % with respect to PSf), and the characteristics of the blended membranes were compared to neat PSf membrane.

## 2. Materials and Methods 

### 2.1. Materials

**Figure 1:**
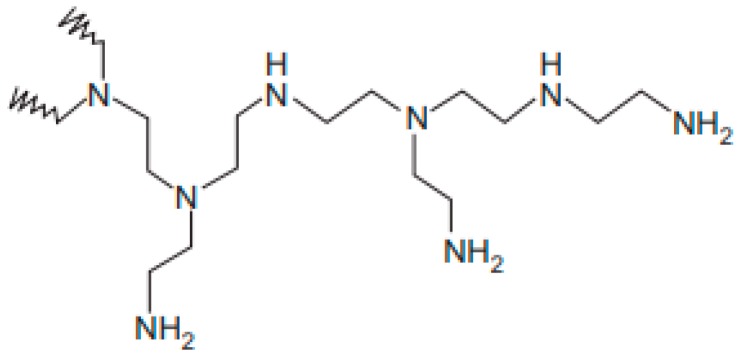
Chemical structure of Lupasol.

Polysulfone (PSf, MW 35000 Da) in transparent pellet form, benzoyl chloride (99% purity), 1-methyl-2-pyrrolidone (NMP, ACS) were purchased by Sigma-Aldrich (Madrid, Spain) and used without any further purification. Lupasol G20 Polyethylenimine (MW 1300 Da, charge cationic density 16 meq/g TS, N:C ratio = 1/2) was kindly provided by BASF (Tarragona, Spain). Its structure is reported in Figure 1. Distilled water was used as coagulation bath in membrane preparation. Hollytex non-woven made of polyester with density 34 g/m^2^ was acquired in STEM and used as support in the CO_2_ module. Extra pure potassium hydroxide in pellets (Scharlab, Barcelona, Spain) was dissolved in deionized water to prepare absorptive solutions.

#### 2.1.1. Additive Preparation

**Scheme 1:**
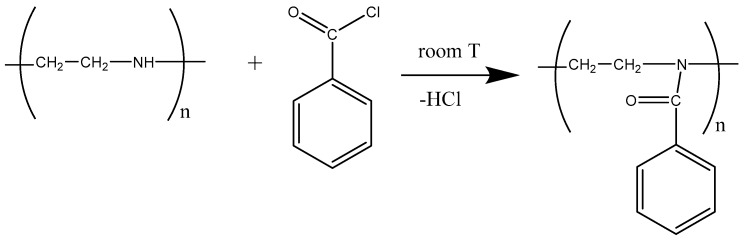
Reaction between Lupasol G20 and benzoyl chloride.

Lupasol G20 was chemically modified, in order to reduce its water solubility, as schematized for the secondary amine groups in Scheme 1.

5.97 g (0.042 mol) of benzoyl chloride were added dropwise to 10 g of Lupasol G20 (ratio chloride/cationic groups = 0.25). The reaction was performed at 25 °C under magnetic stirring for two hours; the reaction product was washed several times with distilled water under stirring and finally decanted, yielding mG20, an oily product which was vacuum dried at room temperature for 24 h. ^1^H NMR (CD_3_OD, TMS, δ, ppm): 6.8–7.9 (m, Ar), 2.2–4.2 (m, N–CH_2_–CH_2_–N).

#### 2.1.2. Membrane Preparation

As seen in Table 1, all membranes were prepared by phase inversion precipitation process in ambient conditions. Specific amount of polymer was dissolved in *N*-Methylpyrrolidone (NMP) to obtain 20 g of solution in which mass ratio between total polymer content and solution was maintained constant at 20 wt %. Resulting mixtures were stirred for 48 h and left overnight for degasification and sonicated for 30 min before the membrane preparation. Solutions were cast on a glass support by using casting knife (knife thickness = 250 μm), and immediately immersed into a coagulation bath containing a non-solvent, where the membranes precipitated as a result of the exchange between solvent (NMP) and non-solvent (water). Prepared flat sheet membranes were taken out from coagulation bath, washed with distilled water, and left overnight to dry in air.

### 2.2. Membranes Characterization

^1^H NMR spectra of mG20 in deuterated methanol (CD_3_OD) were recorded at 400 MHz on a Varian Gemini 400 spectrometer (Varian Inc., Palo Alto, CA, USA). A pulse delay time of 5 s was used. The solvent peak was taken as the reference, and the chemical shifts were given in parts per million from TMS (Tetramethylsilane) with the appropriate shift conversions. 

The membranes morphology was characterized by Environmental Scanning Electronic Microscopy using a FEI Quanta 600 (ThermoFisher Scientific, Waltham, MA, USA). The samples were cryofractured into liquid nitrogen and then fixed on the support suitable for the cross-section analysis. For detection of potassium carbonate, a back scattered electron detector (BSED) was employed.

Images obtained from ESEM were analyzed with ImageJ and IFME software [12] to obtain information about membrane thickness and pore size with 20–30 measurements per sample. 

The overall membrane porosity (ε), defined as the volume of the pores divided by the total volume of the membrane, was determined from the bulk and the polymer density using a method based on the density measurements according to the following equation:

(1) $$\varepsilon \;=\;\left(1-\frac{\rho_{m}}{\rho_{p}}\right)100\%$$

where *ρ*_p_ corresponds to the polymeric material density, which is a weighted average of polusulfone (1.24 g/cm^3^) and lupasol (1.08 g/cm^3^) density, and *ρ*_m_ to the membrane density. *ρ*_m_ is calculated by determining the relationship between mass and volume of 1 cm^2^ of membrane.

The membrane surface morphology and roughness were determined by Atomic Force Microscopy (AFM) and images obtained were analyzed with WsxM software [13]. The membrane surface roughness parameters of the membranes were expressed in terms of root mean square of the Z (*R*_q_).

Water contact angle of the membranes was determined by Dataphysics PCA 15EC. A 3 μL droplet of Milli-q water was placed on the surface of the membrane and the contact angle was calculated from a digital image by SCA software (DataPhysics, Fildertstadt, Germany) included in the apparatus. Contact angle values were taken as the average of three measurements. 

Water uptake tests were performed on previously dried membranes (size: 1.5 × 3 cm^2^). The membranes were dried at 60 °C for 72 h, weighed, soaked in deionized water for 24 h at room temperature, and reweighed. The water uptake (WU) was calculated as:

(2) $$WU\left(\%\right)=\frac{W_{wet}-W_{dry}}{W_{dry}}\;\times 100$$

The glass transition temperature of neat PSf membrane and blended membranes was determined by Differential Scanning Calorimetry by means of a Mettler DSC-821e, calibrated using an in standard (heat flow calibration) and an In-Pb-Zn standard (T calibration). Samples of approximately 5 mg were tested in aluminium pans with a pierced lid in N_2_ atmosphere with a gas flow of 50 mL/min. Tests were performed in dynamic mode at a heating or a cooling rate of 10 °C/minute in cycle: the first heating was from 0 to 200 °C, followed by cooling to 0 °C; the second heating was to 200 °C. *T*_g_ was determined as the inflection point in the heat flow signal step from the second heating scan. 

**Figure 2:**
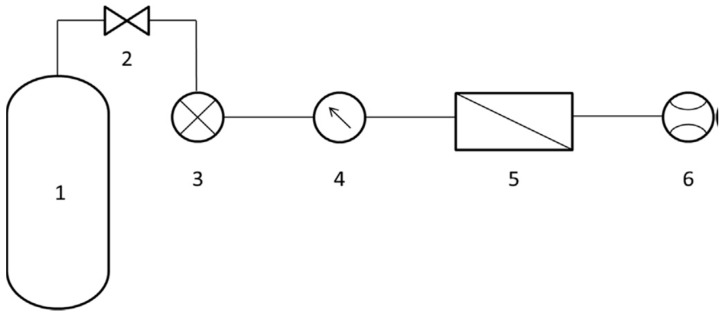
Permeability system: (**1**) CO_2_ gas bottle, (**2**) valve, (**3**) pressure transducer, (**4**) manometer, (**5**) steel module containing membrane, (**6**) gas flow meter.

Gas permeation tests were carried out using a self-made, stainless-steel, dead-end apparatus containing a membrane to measure the carbon dioxide permeance. The effective membrane area in the module was 12.6 cm^2^. Figure 2 shows a schematic representation of the system used in the process. Pure CO_2_ or N_2_ was injected into the module. Permeated gas flow was measured at 1, 2, and 3 bar, respectively, using a precision gas mass flow controller (Alicat Scientific MC-500SCCM-D, Tucson, AZ, USA). 

Finally, a mass transfer coefficient k (mol/s·m^2^·bar) was calculated following the equation:

(3) $$k=\frac{n}{A\cdot \Delta P}$$

where *n* stays for mass transfer (mol/s), *A* is membrane area (m^2^), and Δ*P* driving force pressure gradient (bar).

The solubility of CO_2_ into mG20 additive was studied by measurements of the pressure decay using the system described in Figure 2, with a small modification: the gas flow meter was removed, allowing the membrane-containing module to be closed tightly. Analyzed material was placed in the module and the valve 2 was open to introduce carbon dioxide into the system. When the pressure reached 200 kPa, the valve (2) was closed and pressure decrease was measured. The solubility was calculated according to Equation (4):

(4) $$n=\frac{\left(p_{i}-p_{f}\right)V}{RT}$$

where *n* is the adsorbed moles of CO_2_, *p_i_* and *p*_f_ are the initial and final pressures (Pa), respectively, *V* is gas volume of gas (m^3^), *R* is gas constant (8.314 J/K/mol), and *T* (K) is temperature. The solubility coefficient, *S*_CO2_, expressed in (m^3^ (STP)/m^3^ polymer atm) units, which are commonly used when referring to the solubility of gases, is calculated, respectively, by:

(5) $$S_{CO2}=\frac{V_{CO2m}\left(STP\right)}{V_{m}p_{established}}$$

where *V*_CO2m_ (STP)(m^3^) is the volume of CO_2_ corresponding to *n*_CO2_ at STP conditions (1.013 × 10^5^ Pa, 273.15 K), *V*_m_ is the analyzed material volume [m^3^] and *p*_established_ (atm) is the pressure measured at equilibrium conditions. CO_2_ absorption test was performed in which prepared membranes (9 cm^2^) were placed in a home-made module as reported in [7] and used for CO_2_ absorption test conducted in a gas–liquid membrane contactor. A volume of 200 mL of 0.64 KOH solution was employed as liquid absorbent in contact with the bottom surface of the flat sheet membrane. The liquid absorbent was tested within a range of 35–228 mL/min and the experiments were performed for 1 h with continuous stirring. Bottom surface of the membrane was in contact with absorbent solution while the top surface was exposed to air by holes in the top side of the module. All experiments were carried out in room conditions (25 °C and 1.013 × 10^5^ Pa), and each one was repeated three times to verify the reproducibility. After 1 h, samples were collected and examined for CO_2_ content. 

To determine the absorbed CO_2_ amount, carbon dioxide ion selective electrode (Thermo Scientific connected with Thermo Scientific Orion Dual Star pH/ISE Benchtop meter) was used. Diluted sample (1 mL sample + 45 Milli-q water) was mixed with CO_2_ buffer (5 mL) to adjust the pH to the electrode working pH at 4.8–5.2. The apparatus was set to auto read mode and the CO_2_ concentration in sample was measured. The stable value of CO_2_ flux (mol/m^2^·s) in the membrane contactor was calculated according to the following:

(6) $$J_{CO2}=\frac{Q_{i}\cdot M_{CO2}}{A}\cdot 1000$$

where *Q_i_* is the absorbent flow rate (m^3^/s), *M_CO2_* is CO_2_ concentration in the absorbent (mol/L) obtained from the measurements, and *A* is the area of the flat sheet membrane (m^2^).

## 3. Results and Discussion

As previously stated, commercial Poly (ethylene imine) (PEI), Lupasol G-20, can be a suitable candidate for improving CO_2_ sorption in PSf-based contactors when blended with Polysulfone, thanks to its high content of basic nitrogen. Nevertheless, it is highly soluble in water and this prevents its use in membrane preparation by phase inversion in DMF/water as well as in the final CO_2_ capture device, which contains a basic aqueous solution in contact with the membrane. For this reason, Lupasol G-20 was chemically modified with benzoyl chloride as described in the experimental section to reduce water solubility. As a consequence of this reaction, the number of hindered amines should be increased, improving the carrier capability of this additive.

^1^H NMR of modified Lupasol (mG20) in deuterated methanol, as seen in Figure S1 in supplementary material, showed two groups of broad, partially overlapped signals in the regions 6.8–7.9 (aromatic protons) and 2.2–4.2 (ethylenic protons adjacent to nitrogen atoms), which confirmed that the reaction successfully occurred. According to Lupasol G20 initial composition, the ratio N:C is 1:2; therefore, each ethylene group approximately corresponds to one nitrogen atom. From the integration of the aforementioned signals, we achieved a rough estimation of the number of aromatic groups versus ethylene groups, that is, the aromatic groups/nitrogen atoms content, which resulted in 19%. The amount of benzoyl chloride for the reaction was 25% with respect to the cationic charge density of Lupasol G20; accordingly it can be concluded that chemical modification of Lupasol G20 occurred to a good extent. The achieved degree of modification was sufficient to reduce polymer solubility in water: mG20 was obtained after precipitation and washing in water.

Afterwards, mG20 was blended with PSf in different amounts, namely 2, 5, 10, and 20 wt %, as seen in Table 1, by dissolving both polymers in N-methylpyrrolidone (20 wt % polymer/solvent) as described in the experimental section. Next, polymeric solutions were casted and immersed into a coagulation bath containing water, finally yielding flat sheet membranes (L2–L20), whose morphology was characterized by means of environmental scanning electron microscope (ESEM).

**Figure 3:**
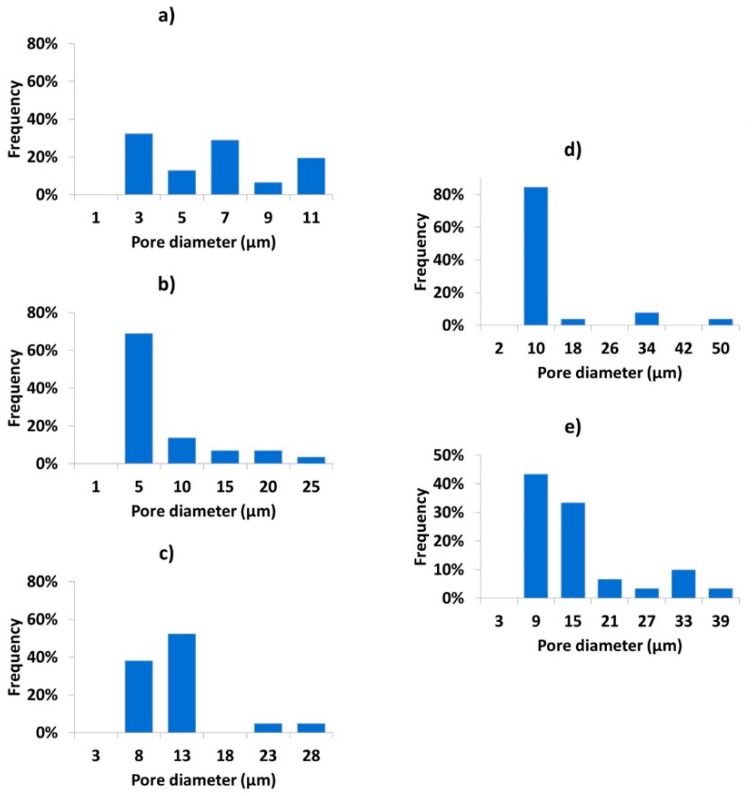
The pore size distribution histograms of membranes: (**a**) L0; (**b**) L2; (**c**) L5; (**d**) L10; (**e**) L20.

Membrane thicknesses were determined by ESEM micrographs by means of ImageJ and IFME softwares, and are reported in Table 1. Porosity was calculated from the densities. Additionally, the pore size distribution was also determined by ESEM images and further analysis with the previously mentioned software; the obtained histograms are included in Figure 3. As can be seen in Figure 3a, in the case of L0, 90% of pores are homogeneously distributed at 3–11 µm, while in blended membranes, 80% of measured pores lie in the range 8–15 µm, i.e., the pore diameter increases when mG20 content is increased, though the number of pores decreases. For instance, while in case of the L2 membrane, 70% of pores diameter gave a value of 5 µm, in the L20 membrane, 70% of pores ranges were between 9 and 13 µm, and huge pores, with a diameter above 30 µm, were observed.

**Figure 4:**
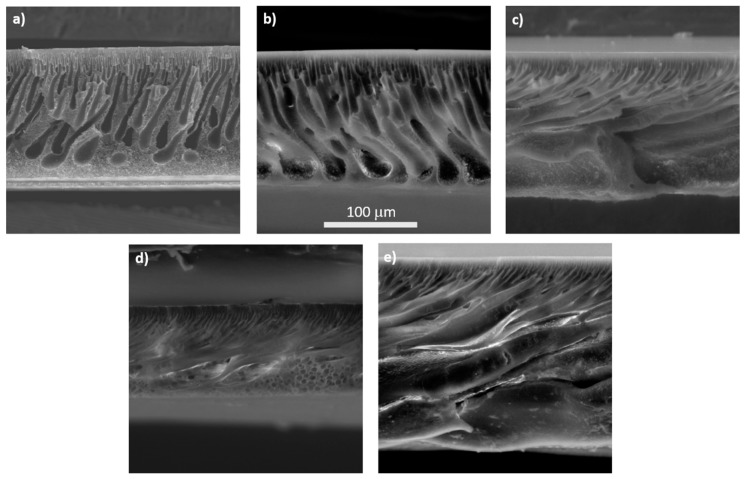
Environmental scanning electron microscope (ESEM) micrographs of the cross-section of PSf-mG20 membranes: (**a**) L0; (**b**) L2; (**c**) L5; (**d**) L10; (**e**) L20.

Figure 4b–e show the cross-section images of L2, L5, L10, and L20 membranes. For sake of comparison, the image of pure PSf membrane (L0) is also reported, as seen in Figure 4a.

Figure 4a shows that the neat PSf membrane (L0) exhibits a finger-like structure on top of a spongy bulk matrix. A very similar morphology can be found for L2. On the other hand, when mG20 additive amount was increased, membrane morphology progressively changed: as seen in Figure 4c, in L5, finger-like pores decreased but a considerable amount of voids remained evident; L10 shows an evident spongy layer with a much denser layer, as seen in Figure 4d. In the case of L20, morphology is definitely denser, with some residual porosity on the top side, as seen in Figure 4e. This change is also shown by the trend observed for porosity values, which are related to the void contents, as explained in the experimental part. They initially increased with increasing mG20 content, reaching the highest value for L5; subsequently, they decreased, with a minimum for L20. This confirms that membrane morphology becomes denser at higher mG20 contents. Membrane morphology is predominantly affected by the precipitation rate during the phase inversion process. Fast precipitation is determined by fast penetration of the non-solvent into the polymeric solution and leads to highly asymmetric structures, with fingerlike macrovoids; slower coagulant penetration, and consequently slower precipitation, produce sponge-like and more symmetrical structures. Thus, the more affine polymer and the coagulant are, the slower precipitation will be, and the more symmetrical structures can be expected. In our case, mG20 additive is a relatively hydrophilic, polar polymer; when blended with PSf, it contributes to a decrease in the precipitation rate of the polymeric solution. This effect is more evident in L20, which contains the highest mG20 amount and exhibits the densest structure and the lowest porosity of the whole series. At low mG20 content, the effect of this component seems to be a very limited change of the solution thermodynamic stability, with consequent similar precipitation rates, and final membrane morphologies which strongly resemble the one of pure PSf; on the other hand, when mG20 amount increases, then the solution thermodynamic stability seems to be enhanced, leading to slower precipitation rates and resulting in final morphologies remarkably different with respect to neat PSf. In addition, it should be taken into account that the presence of an additive not only modifies the thermodynamic stability of the polymeric solution but can also alter its viscosity, which can determine a change in the phase inversion process. 

**Figure 5:**
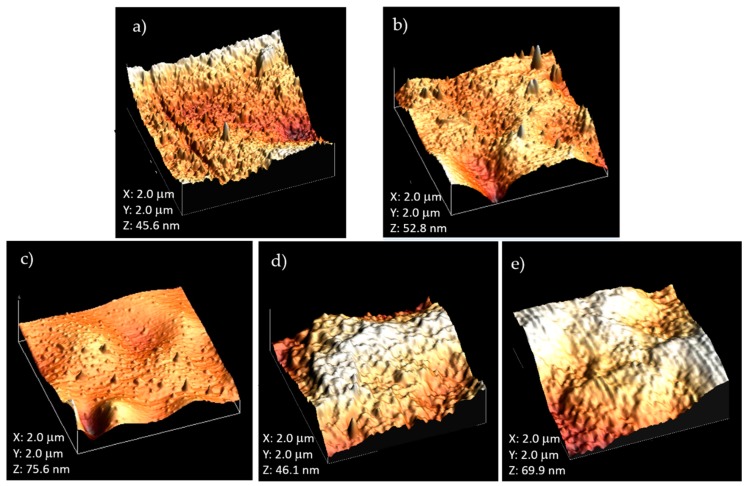
Atomic Force Microscopy (AFM) topographic images of bottom side of PSf-mG20 membranes: (**a**) L0; (**b**) L2; (**c**) L5; (**d**) L10; (**e**) L20.

The obtained membranes are to be used in a device wherein the bottom surface is in direct contact with an aqueous absorbent solution. Therefore, PSf-mG20 membranes were characterized in terms of surface morphology, water contact angle, and water uptake to establish whether blending with mG20 could eventually affect their wettability with respect to neat PSf. Figure 5a–e report the AFM topographic image pattern of bottom side of L0–L20 membranes; Table 2 reports their RMS roughness as well as the values of the water contact angle and water uptake.

As seen in Table 2, blending with mG20 determines an increase of RMS roughness of bottom side, i.e., the higher mG20 amount, the rougher the bottom membrane surface. As for membrane wettability, water contact angle decreases with an increase of mG20 content, as can be expected based on its higher hydrophilicity with respect to PSf; moreover, this effect is more evident on the bottom of the membrane, which suggests that mG20 could be more concentrated on this side.

**Figure 6:**
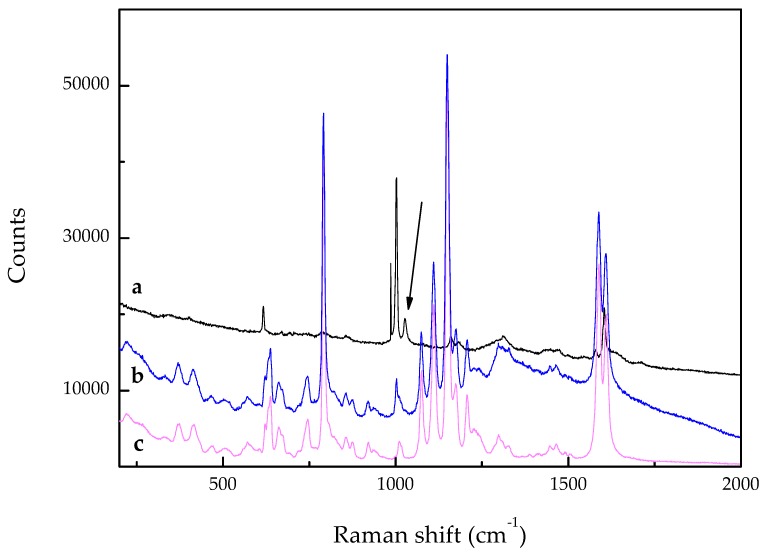
Raman spectra between 200 and 2500 cm^−1^ of: (**a**) mG20; (**b**) Top surface of L20; (**c**) L0. The arrow indicates the band at 1030 cm^−1^.

To determine the distribution of mG20 additive in PSf membrane, we checked Raman absorption across their section between 200 and 2500 cm^−1^ at different depths, starting from the top side moving towards the bottom side. Figure 6 shows Raman spectra of recorded on the top of L20 membrane, of neat mG20 additive and L0 (neat PSf) membrane, respectively. The only band which can be attributed exclusively to the additive and it is not overlapped with other PSf bands is that at 1030 cm^−1^, as seen in Figure 6a, attributed to the C–N stretching of secondary and tertiary aliphatic amines [14]. This band can be guessed as a shoulder also in the Raman spectra of L20 membrane, as seen in Figure 6b, which contains the highest amount of additive. As seen in Figure S2 in Supplementary Material, mG20 seems to be present throughout the whole membrane cross-section since the shoulder at 1030 cm^−1^ can be observed in the spectra of top, center, and bottom of the membrane. Unfortunately, when the Raman spectra of L10 membrane were observed, as seen in Figure S3 in the Supplementary Material, this band was not visible, probably due to the lower amount of mG20 in this sample. The same was also found in the case of L2 and L5 membranes. Therefore, we could not check the actual distribution of mG20 in L2–L10 membranes.

We also studied water uptake (WU) of L0–L20 membranes. As shown in Table 2, the presence of mG20 additive remarkably increases water uptake of these systems with respect to neat PSf; however, only 2 wt % additive determines 6% WU in comparison with 0.4% of neat PSf (L0). Finally, L20 membrane, with the highest mG20 content, exhibits two orders of magnitude higher WU than L0. This effect can be attributed to the high hydrophilicity of mG20 additive and could compromise the dimensional stability and performance of the membranes, thus representing a drawback in the use of these composite membranes in the final CO_2_ capture device. Penetration of the liquid into the membrane pores negatively affects absorption flux since, in this case, gas diffusivity in the liquid phase is far lower than in the gaseous phase.

Absorption performance of composite membranes was evaluated for potassium hydroxide solution in a flat sheet membrane contactor (FSMC) in a cross flow module at different liquid flow rates. Results are shown in Figure 5 as carbon dioxide flux versus absorbent flow rate.

**Figure 7:**
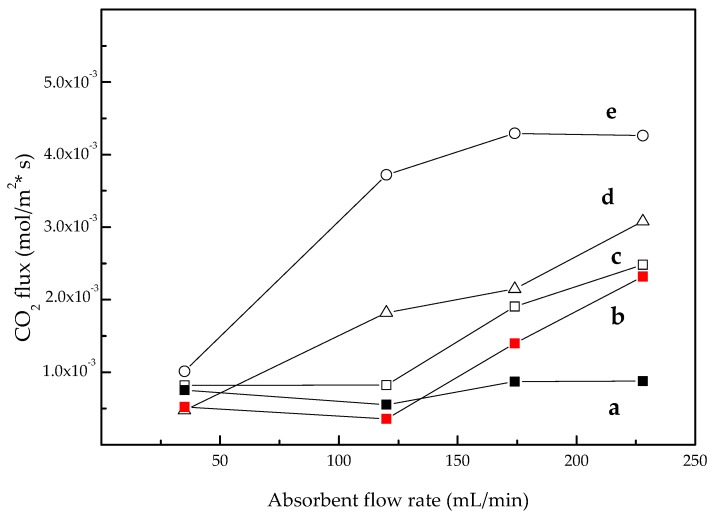
CO_2_ absorption flux into L0–L20 membranes as a function of liquid absorbent flow rate. (**a**) L20; (**b**) L10; (**c**) L0; (**d**) L2; (**e**) L5.

Results show that, at the lowest absorbent flow rate (35 mL/min), performances of the membranes are very similar as regards CO_2_ flux, as seen in Figure 7. In the mass transfer process between gaseous and liquid streams through a porous membrane, resistance can be split into three terms: liquid side, gas side, and membrane mass transfer resistances [15]. The effect of gas side resistance can be neglected because we used ambient air as the CO_2_ source, thus gas concentration was constant in all our experiments. At low liquid flow rate, the resistance term related to the liquid side mainly affects the total mass transfer resistance. This determines very similar gas absorption flux at low liquid absorbent flow rate, as shown in Figure 7. Nevertheless, on increasing absorbent flow rate (120–228 mL/min), remarkable differences can be found, which can be attributed to the different mG20 additive content; in particular, the behavior of the membranes with lower additive amount (L2 and L5) was more efficient than neat PSf, with L5 having the best performance. On the other hand, the presence of higher amounts of mG20, namely 10 and 20 wt %, seems to be detrimental to CO_2_ capture when compared with neat PSf.

With an increase in the liquid flow rate, the liquid side mass transfer resistance is reduced and eventually, above a certain flow rate, the mass flux is predominantly influenced by the membrane resistance. When this occurs, the plot of absorption flux versus liquid flow rate levels off. In our case, this situation is clearly shown by the L5 and L20 membranes; however, we will try to explain the behavior of the whole series in terms of membrane mass transfer resistance. L0–L20 membranes differ as far as chemical composition and morphology are concerned: therefore, different factors should be taken into account. First, the presence of mG20 additive determines a remarkable increase of CO_2_ solubility into the polymeric membrane. CO_2_ solubility into neat PSf membrane was determined as 4.79 ± 0.01 m^3^ STP/m^3^ atm [7]; in contrast, this value rose to 21.3 m^3^ STP/m^3^ atm for neat mG20 additive. On the other hand, the presence of the additive strongly affects membrane morphology, reducing porosity, and void contents, and is thus detrimental to CO_2_ diffusivity. Another factor, previously mentioned, is the strong increase in wettability and water uptake deriving from mG20 addition, which, in turn, can help the penetration of the liquid phase into the membrane, provoking membrane clogging, and negatively affecting gas diffusivity. Another point that could have some influence on membrane performance is the plasticizing effect of mG20 on PSf; for neat PSf (L0) membrane, a glass transition temperature of 184 °C was found, while this value reduced to 173 °C for the L20 membrane. Glass transition is strictly related to the polymer free volume, which also has a role in gas transport through polymeric membranes [16,17]

Permeation in glassy polymers has been modeled in terms of diffusion solution. The dual mode sorption model [18,19] assumes that a polymer consists of a continuous chain matrix and microvoids frozen in the matrix. These voids are caused by the unrelaxed volume of polymers in the glassy state. Therefore, the dual mode sorption model makes reference to two terms: the former based on Henry’s law of solubility, i.e., dissolution of the permeant in the continuous chain matrix; the latter is a Langmuir-type term related to permeant sorption in microvoids. Therefore, in our case, it is reasonable to suppose that the addition of mG20 determines two contrasting effects. On the one hand, increased amounts of the additive enhance CO_2_ solubility into the polymeric matrix; nevertheless, this also provokes a decrease in microvoids content, as shown by ESEM analysis. Importantly, mG20 greatly increases water uptake and therefore 

**Figure 8:**
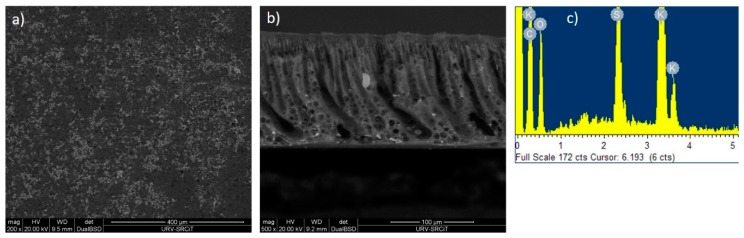
ESEM micrographs with BSED of L2 membrane after four experiments in a flat sheet membrane contactor: (**a**) bottom surface; (**b**) cross-section; (**c**) X-ray analysis (EDX) analysis of the white spots seen in the micrographs.

KOH solution penetration from the bottom side of the contactor, with consequent membrane pores clogging. L5 membrane shows the best performance in the whole series, suggesting that this additive amount (5 wt %) represents the best compromise between these two opposite effects. It was reported that total pore volume and specific surface of nanoporous shale decreased after CO_2_ adsorption, and that this phenomenon can be interpreted in terms of physisorption, associative chemisorption, and dissociative chemisorption [20]. The first is related to the adsorption of carbon dioxide molecules in the organic pores that produces a variation in the shale surface energy, which, in turn, produces a swelling phenomenon; as a consequence, the pore volume decreases, pores are blocked and CO_2_ is trapped inside the structure. This phenomenon has been modelled and was found to be relevant in the case of pressure higher than 0.5 MPa [21]; however, in our case, experiments were performed at CO_2_ atmospheric pressure. An interesting parallel experiment could be performed with associative chemisorption of carbon dioxide at shale mineral surfaces or pores: in conditions of high pressure and temperature, CO_2_ and H_2_O can react with minerals, thus generating new products, such as carbonates, which can cover the pore walls and cause geochemical and morphological changes to the shale. Consequently, the pore may be blocked and the pore volume decreases. In our case, penetration of absorber KOH solution into the blended membrane was expected to induce the precipitation of K_2_CO_3_ that could block membrane pores in a similar way. As a matter of example, Figure 8a,b show the bottom surface and cross-section ESEM micrographs taken with BSED of L2 membrane, respectively, after four experiments in the flat sheet membrane contactor. In contrast, Figure 8c shows the results of energy dispersive X-ray analysis (EDX) of the white spots observed in the shown micrographs.

Figure 8a,b demonstrate the presence of white crystals both on the bottom surface of the membrane in direct contact with KOH solution and on the bottom side of the membrane cross section. As seen in Figure 8c, EDX analysis revealed that these crystals could be formed by potassium carbonate. Therefore, these results suggest that, even in the case of L2 membrane, the penetration of KOH solution from the contactor into the membrane can induce K_2_CO_3_ precipitation that may eventually block the pores. This effect is expected to be more pronounced in blended membranes with much higher water uptake and, consequently, more efficient penetration of KOH solution, i.e., L10 and L20, and could drastically reduce membrane performances. 

Permeability tests were performed to calculate the mass transfer coefficients, k, for the L0–L20 membranes series. K was calculated from CO_2_ as well as from N_2_ permeation to verify the role of chemical composition in the gas permeability of these membranes.

**Figure 9:**
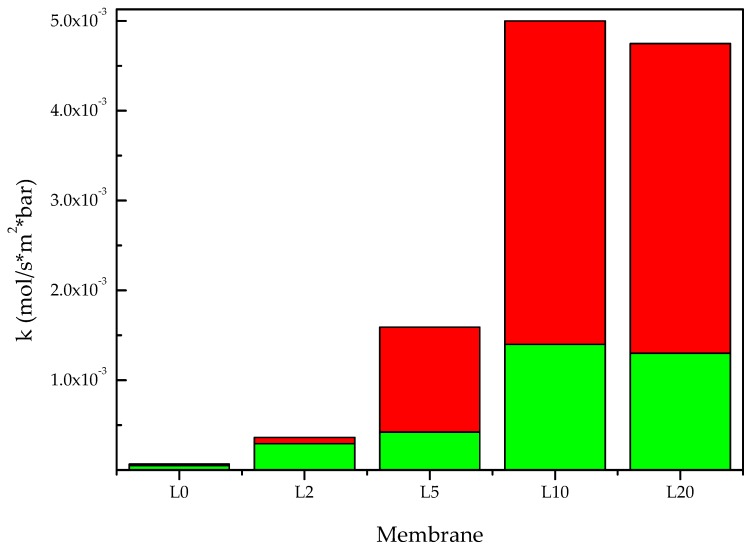
Mass transfer coefficient of L0–L20 membranes relative to: N_2_ (**green bar**); CO_2_ (**red bar**).

Figure 9 reports the mass transfer coefficient k calculated from CO_2_ and N_2_ permeation through L0–L20 membranes.

Figure 9 shows that k has an increasing trend with increasing amount of mG20: the addition of just 2 wt % mG20 determines an order of magnitude higher mass transfer coefficient for both CO_2_ and N_2_ permeation, despite the similar morphology of L0 and L2 membranes. Nevertheless, the effect of mG20 is much more pronounced for carbon dioxide than for nitrogen, when the full composition range is examined. In addition, the N_2_ mass transfer coefficient is always lower than CO_2_ mass transfer coefficient for L2–L20 systems despite having practically the same value in L0 which contains no additive. This suggests that the permeation of these gases does not only depend on the ratio between the pores of the membranes and molecular size of gases, but also on the chemical composition, that is, it is not exclusively governed by physical parameters. Results suggest that carbon dioxide permeation was enhanced thanks to its favorable interaction with the amines contained in mG20, provided that, for instance, the Van der Waals radius of nitrogen is 1.95 Å, while that of carbon dioxide is 5.331 Å in the direction of the oxygen atoms and 3.033 Å in the normal direction [21,22]. 

Therefore, the addition to PSf membranes of relatively low amounts of a high molecular weight additive such as mG20, which contains a big amount of basic amine groups, is effective in improving CO_2_ permeability in the gas phase.

## 4. Conclusions

We tested the use of a high molecular weight additive, mG20, obtained by chemical modification of a commercial Polyethylenimine, to improve the performance of PSf membranes in membrane contactors for CO_2_ capture due to the presence of basic nitrogen atoms. The amount of blended mG20 was in the range of 2–20%. We found that the presence of the polymeric additive determined a change in the membrane morphology: porosity was increased up to 5 wt % mG20, but decreased upon further mG20 addition, giving denser membranes. Blending with the hydrophilic additive produced membranes with rougher surfaces, higher water contact angles and water uptake, as expected. The presence of mG20 also improved the mass transfer coefficient for both N_2_ and CO_2_, but the effect was much more marked for the latter, revealing an increase in carbon dioxide permeability as a consequence of blending with the additive. On the other hand, when the application of these membranes in the final CO_2_ capture device is considered, we found that, when mG20 amount was higher than 5 wt %, increased water uptake and eventual K_2_CO_3_ precipitation provoked membrane pores clogging and negatively affected CO_2_ permeability. This effect was more evident when higher liquid flow rates were used in the contactor.

In conclusion, the performance of PSf membranes in carbon dioxide capture device can be greatly improved by blending with mG20 amount as low as 5 wt % since this composition represents the best compromise between higher membrane affinity for CO_2_, induced by the presence of basic nitrogen atoms and the changes in morphology, wettability, and water uptake, which are detrimental at higher additive amounts. The study of durability and consequent performance variation of these composite membranes is currently under study and will be the subject of a forthcoming paper.

## Figures and Tables

**Table 1:** Composition and morphological characteristics of the investigated membranes.

Membrane	mG20 Content (wt. %)	Thickness (µm)	Porosity (%)
L0	0	123 ± 4	74.3
L2	2	139 ± 3	79.0
L5	5	151 ± 6	81.8
L10	10	162 ± 2	75.8
L20	20	196 ± 3	69.2

**Table 2:** RMS roughness of bottom side, water contact angle (CA) and Water Uptake (WU) of PSf-mG20 membranes.

Membrane	RMS Roughness (nm)	Top CA (°)	Bottom CA (°)	WU (%)
L0	7.5	87 ± 3	86 ± 4	0.40 ± 0.05
L2	6.1	83 ± 1	72 ± 4	6.0 ± 0.6
L5	8.2	79 ± 2	73 ± 3	45 ± 5
L10	10.5	77 ± 2	70 ± 1	142 ± 20
L20	15.8	77 ± 1	65 ± 1	274 ± 17

## Supplementary Materials

The following are available online at https://www.mdpi.com/2073-4360/11/10/1662/s1, Figure S1: ^1^H NMR spectrum in CD_3_OD of mG20; Figure S2: Raman spectra between 200 and 1500 cm^−1^ performed across the section of L20; Figure S3: Raman spectra between 200 and 1800 cm^−1^ of: (a) neat mG20; (b) L10, top surface; (c) L10, bottom surface.
