# Supplementary material for: Polymer Blends for Improved CO2 Capture Membranes
Source: Polymers (Basel). 2019 Oct 12;11(10):1662. doi: 10.3390/polym11101662 (PMC6835398; doi:10.3390/polym11101662)
Supplement: Supplementary file 1 [file polymers-11-01662-s001.pdf]

Article

Polymer Blends for Improved CO_2 Capture Membranes

Alireza Zare [1], Lorenza Perna [1,4], Adrianna Nogalska [2,3], Veronica Ambrogi [4], Pierfrancesco Cerruti [5], Bartosz Tylkowski [2,3], Ricard García-Valls [1,2,3], and Marta Giamberini [1,*]

[1] Department of Chemical Engineering, Universitat Rovira I Virgili, Av. Països Catalans, 26, 43007 Tarragona, Spain; alireza.zare@urv.cat (A.Z.); lorenza.perna@hotmail.com (L.P.); ricard.garcia@urv.cat (R.G.-V.)

[2] Chemistry Technology Centre of Catalonia (CTQC), C/Marcel·lí Domingo, 43007 Tarragona, Spain

[3] Eurecat, Centre Tecnològic de Catalunya, C/Marcel·lí Domingo, 43007 Tarragona, Spain; adrianna.nogalska@ctqc.org (A.N.); bartosz.tylkowski@ctqc.org (B.T.)

[4] Department of Chemicals, Materials and Production Engineering, University of Naples Federico II, Piazzale Tecchio 80, 80125 Naples, Italy; ambrogi@unina.it

[5] Institute of Polymers, Composites and Biomaterials, National Research Council, Via Campi Flegrei 34, 80078 Pozzuoli, Italy; cerruti@ipcb.cnr.it

* Correspondence: marta.giamberini@urv.cat; Tel.: +34 977558174

Received: 10 September 2019; Accepted: 10 October 2019; Published: date

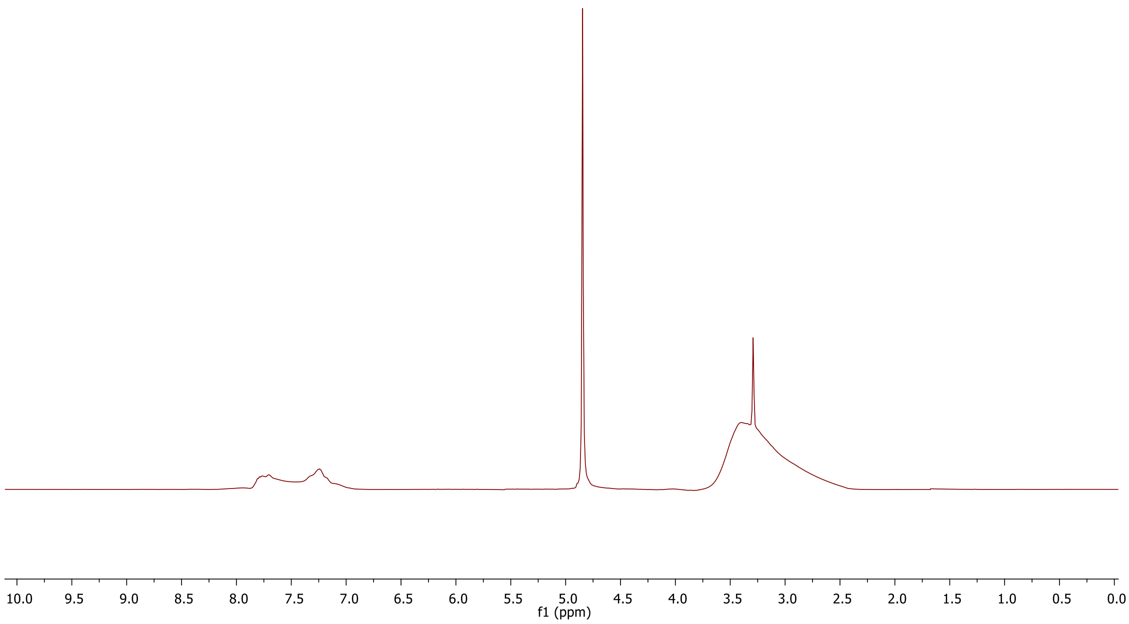

Figure S1. ^{1}H NMR spectrum in CD_3OD of mG20.

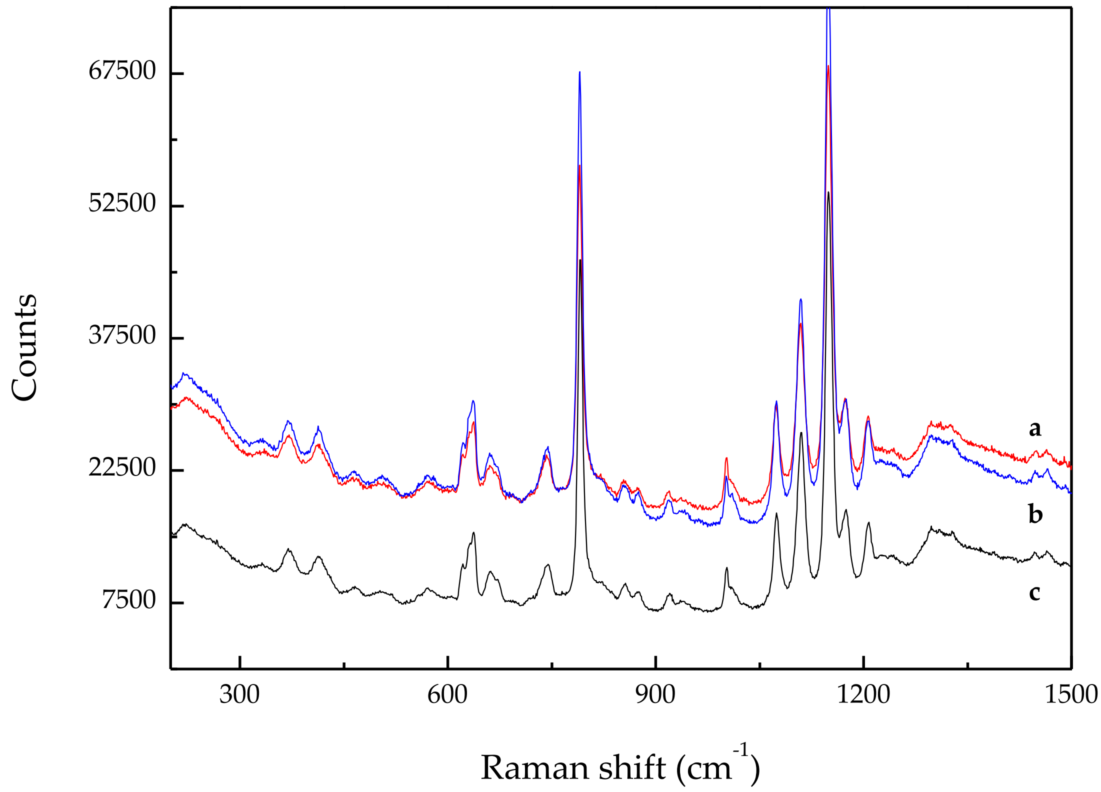

Figure S2 Raman spectra between 200 and 1500 cm^{-1} performed across the section of L20 : (**a**) top; (**b**) center; (**c**) bottom surface.

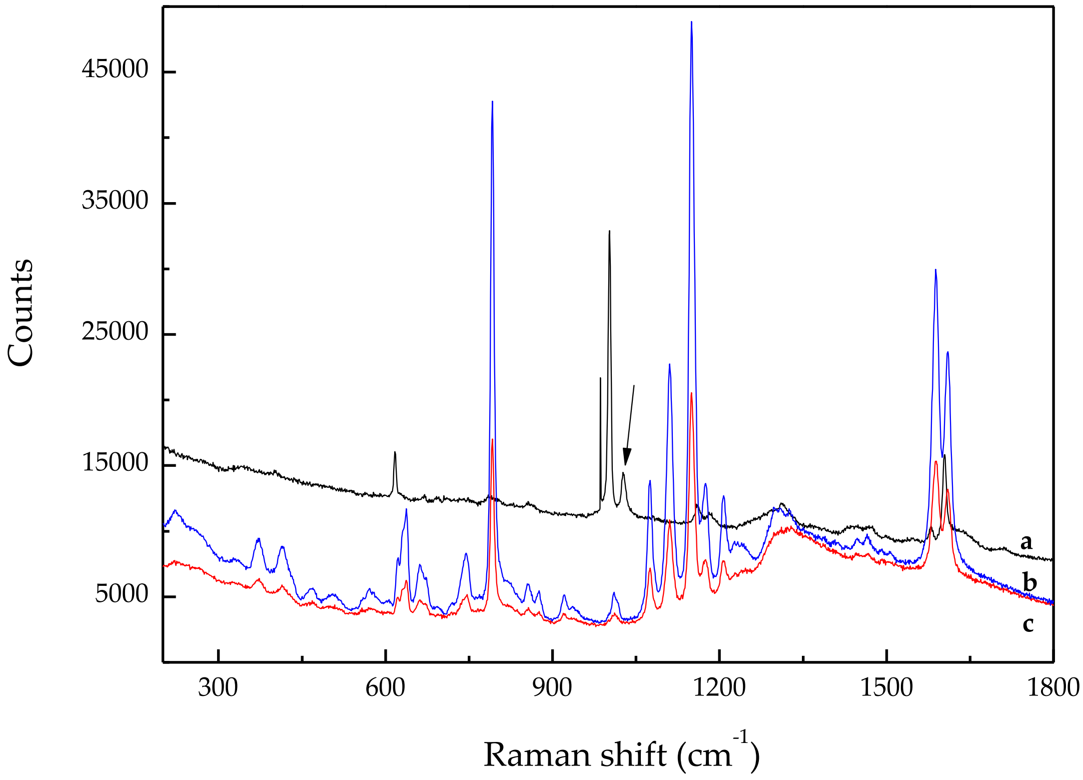

Figure S3 Raman spectra between 200 and 1800 cm^{-1} of: (a) neat mG20; (b) L10, top surface; (c) L10, bottom surface. The band at 1030 cm^{-1} is highlighted.
